# Respiratory function of people with amyotrophic lateral sclerosis and caregiver distress level: a correlational study

**DOI:** 10.1186/1751-0759-6-14

**Published:** 2012-06-21

**Authors:** Francesco Pagnini, Paolo Banfi, Christian Lunetta, Gabriella Rossi, Gianluca Castelnuovo, Anna Marconi, Federica Fossati, Massimo Corbo, Enrico Molinari

**Affiliations:** 1 Department of Psychology, Catholic University of Milan, Milan, Italy; 2 NEuroMuscular Omnicentre (NEMO), Fondazione Serena Onlus, Ospedale Niguarda Cà Granda, Milan, Italy; 3 Department of Neuromuscular Disease, Fondazione Don Gnocchi, Milan, Italy; 4 Istituto Auxologico Italiano Istituto di Ricovero e Cura a Carattere Scientifico (IRCCS), Psychology Research Laboratory, San Giuseppe Hospital, Verbania, Italy

**Keywords:** Quality of life, Bio-psycho-social interaction, Amyotrophic lateral sclerosis, Non-invasive ventilation, Health care, Caregivers

## Abstract

**Background:**

Amyotrophic Lateral Sclerosis (ALS) is a rare, fatal neurodegenerative disorder with no curative treatment characterized by degeneration of motor neurons involving a progressive impairment of motor and respiratory functions. Most patients die of ventilator respiratory failure. Caregivers have a great influence on the patient”s quality of life as well as on the quality of care. Home influence of the caregiver on patient care is notable. To date, no study has investigated how psychological issues of caregivers would influence respiratory variables of ALS patients. The study aimed at finding out if there is a relationship between the respiratory function of ALS patients and the level of distress of their caregivers.

**Methods:**

A cross-sectional study was conducted to investigate respiratory issues (PCF and FVC) and the perception of social support of ALS patients. Caregivers filled questionnaires about trait anxiety, depression, and burden of care. Forty ALS patients and their caregivers were recruited.

**Results:**

FVC and PCF were positively related to patient perception of social support and negatively related to caregiver anxiety, depression, and burden.

**Discussion:**

The distress of ALS caregivers is related to patient respiratory issues. The first and more intuitive explanation emphasizes the impact that the patient’s clinical condition has with respect to the caregiver. However, it is possible to hypothesize that if caregivers feel psychologically better, their patient’s quality of life improves and that a condition of greater well-being and relaxation could also increase ventilatory capacity. Furthermore, care management could be carried out more easily by caregivers who pay more attention to the patient's respiratory needs.

**Conclusion:**

Patient perception of social support and caregiver distress are related to respiratory issues in ALS.

## Background

Amyotrophic lateral sclerosis (ALS) is a fatal neurodegenerative disorder affecting motor neurons in the anterior horn of the spinal cord, the brainstem, and the motor cortex. It is clinically characterized by progressive weakness leading to death by respiratory insufficiency within usually three years [1]. However, the progression of the disease is unpredictable, with 10% of patients with ALS living more than ten years [2]. So far it is not possible to stop the progression of the disease.

The incidence of the disease is reported to be between 1.5 and 2.7 per 100,000 population/year (average 1.89 per 100,000/year) in Europe and North America [3].

The onset of the disease rarely involves respiratory muscle [4], but all patients eventually experience respiratory muscle weakness, leading most of them to die from respiratory failure or pneumonia [5]. Pulmonary evaluation is a very important concern in ALS care and management, in a multidisciplinary context [6].

The role of caregivers, typically a spouse or a sibling who lives with the patient and provides him “with the most care and assistance” [7], is very important for ALS care. Previous studies reported that caregivers have a great influence on a patient’s quality of life (QoL), as well as on the quality of care [8,9]. Some caregivers report positive experiences from caregiving, including a sense of giving back to someone who has cared for them [10]. However, being the caregiver of someone with ALS is not simple. Caregivers often experience burden due to personal and social restrictions and to physical and emotional problems [11,12]. They spend much time for day care, sometimes more than 11 hours a day, despite having other chores in the home [13].

Some study has been done about how ALS influences a caregiver’s life [14], but very little information is available about how the caregiver’s life influences ALS. Clinical evidence suggests that caregivers should be involved in care management that involves helping the patient at home. Hence, it is very important to understand how a physician can enhance such assistance.

The loss of a patient’s physical functions seems to be related to the distress experienced by the caregiver [9]. However, to our knowledge, no study of ALS has investigated the interactions between the caregiver’s suffering and their patient’s medical parameters. We studied the hypotheses that caregivers distress could be related to respiratory condition in ALS patients. Thus, we attempted to verify whether or not such a clinical parameter might be correlated with the patient’s perception of received social support.

## Methods

The study included 40 ALS patients (16 females, 24 males), together with their informal caregivers (31 females and 9 males), recruited at NEMO - NEuroMuscular Omnicenter, a clinical service specializing in the treatment and management of neuromuscular disorders with a multidisciplinary approach. A caregiver was defined as someone who lives with and provides the patient “with the most care and assistance” [7]. All patients had been diagnosed with probable or definite ALS according to the revised criteria of El Escorial [15] and had taken Riluzole for over 6 months. The exclusion criteria included the presence of psychopathology in ALS patients or their caregivers before the onset of the illness, verified by a specialized psychologist during the assessment interview. Patient clinical data were assessed by nurses trained in respiratory care, while the psychological characteristics of all subjects were obtained by a specialized clinical psychologist. Every subject gave informed consent to participate in the study. The study design was approved by our hospital’s ethics committee.

Patient assessment consisted of measurement of Peak Cough Flow and Forced Vital Capacity together with the “Social Support” (SS) sub-scale from the McGill Quality of Life (MQoL) questionnaire. Caregivers compiled the Zarit Burden Interview (ZBI), Beck Depression Inventory-II (BDI-II), and State-Trait Anxiety Inventory (STAI).

### Patient assessment

1. The Peak Cough Flow (PCF) test was administered by instructing the patient to inspire completely and cough forcibly thought a face mask attached to a pneumotachograph PCFp (Quark PFT, Cosmed USA Inc.), as this method is the most accurate for the assessment of lower flow range [16].

2. The Forced Vital Capacity (FVC) was measured only in a supine position, according to 1995 ATS standards [17].

3. Perceived social support was assessed with the “Social Support” sub-scale (MQOL-SS) from the McGill Quality of Life questionnaire [18]. It includes two questions with an 11-point likert scale: “I have felt supported (from 0 = not at all to 10 = completely)” and “the world has been… (from 0 = an impersonal unfeeling place to 10 = caring and responsive to my needs”). No cut-off scores are provided, but higher scores indicate a perception of better social support.

### Caregivers assessment

1. The Zarit Burden Interview (ZBI) is a 22-item questionnaire with responses ranging from 0 to 4 and a total score range from 0 to 88 [19]. It is often used in the literature as a measure of caregiver burden. Higher scores indicate heavier burden on the caregiver [20]. The proposed cut-off for severe caregiver burden is 25 [21].

2. The Beck Depression Inventory-II (BDI-II) consists of 21 items to assess the intensity of depression in clinical and normal patients. Each item has a list of four or more statements arranged in increasing severity about a particular symptom of depression (range from 0 = no depression to 63 = severe depression) [22]. Scores ranging from 0 to 13 reflect absent to minimal depression, while scores over 19 are generally considered to signify moderate to severe depression.

3. The State-Trait Anxiety Inventory (STAI) is a self-report assessment device that includes separate measures of state and trait anxiety. We used only the trait form, composed of 20 items. Trait anxiety denotes "relatively stable individual differences in anxiety proneness…" and refers to a general tendency to respond with anxiety to perceived threats in the environment [23]. The overall (total) score for STAI ranges from a minimum of 20 to a maximum of 80; STAI scores are commonly classified as 'no or low anxiety' (20–37), 'moderate anxiety' (38–44), and 'high anxiety' (45–80).

### Data analysis

All analyses were conducted using SPSS - Statistical Package for Social Science, version 13. We investigated correlations (two-tailed) between all variables at T1 using Pearson’s r. Bonferroni correction was used for multiple analyses.

## Results

Patient and caregiver characteristics are reported in Tables 1 and 2. No patients received non-invasive ventilation. The mean ages, respectively, were 61,73 and 55,64 years, while the mean length of illness was 15 months since symptom onset. The mean FVC and PCF scores were under the normal values, indicating limited respiratory problems. A moderate mean level of trait anxiety and a minimal average level of depression were founded in the caregivers.

**Table 1:** Characteristics of patients and caregivers

	**ALS patients (n = 40)**	**Caregivers (n = 40)**
Sex (n,%)		
Female	16 (40%)	28 (70%)
Male	24 (60%)	12 (30%)
Age (Mean, SD)	61,73 (11,5)	55,64 (12,3)
Educational level (n,%)		
Primary school	15 (37,5%)	13 (32,5%)
High school	19 (47,5%)	17 (42,5%)
University	5 (12,5%)	7 (17,5%)
Post-graduate	1 (2,5%)	3 (7,5%)
Mean length of illness (since symptom onset)	15 months	
ALSFRS (mean, SD)	34,87 (7,78)	
Caregiver type (n,%)		
Partner/spouse		33 (82,5%)
Immediate family		7 (17,5%)

**Table 2:** Patient and caregiver mean values

	**Mean (SD)**
ALS Patients	
FVC	86,38 (25,9)
PCF	5,25 (2,29)
MQOL-SS	5,81 (3,01)
Caregivers	
STAI	41,33 (11,19)
BDI-II	9,03 (8,94)
ZBI	17,91 (15,35)

There was a high degree of correlation between all variables (Table 3). Patient FVC and PCF were highly related (r = .585, p < .01).

**Table 3:** **Correlations (pearson’s *r*) between variables**

			**Patient**			**Caregiver**	
Patient		FVC	PCF	MQOL-SS	STAI	BDI-II	ZBI
	FVC	1	.585**	.688**	-.508**	-.606**	-.418**
	PCF	.585**	1	.391*	-.525**	-.622**	-.354*
	MQOL-SS	.688**	.391*	1	−345*	−308*	-.529**
Caregiver	STAI	-.508**	-.525**	−345*	1	.596**	.358*
	BDI-II	-.606**	-.622**	−308*	.596**	1	.430**
	ZBI	-.418**	-.354*	-.529**	.358*	.430**	1

*p < .05; **p < .01.

The relationship between clinical and psychological variables was high. Patient FVC was negatively related to caregiver STAI (r = −.508, p < .01), BDI-II (r = −.606, p < .01) and ZBI (r = −.418, p < .01). The analysis of PCF correlations indicated similar trends (with STAI: r = −.525, p < .01; BDI-II: r = −.622, p < .01; ZBI: r = −.354, p < .05). These correlations remaind high for caregivers with BDI-II scores higher than 18.

The perception of Social Support indicated by patients presented significant correlations with FVC (r = .688, p < .01) and PCF (r = .391, p < .05).

Caregiver ZBI, STAI, and BDI-II were positively related but negatively related to the patients MQOL-SS.

## Discussion

The management of ALS patient’s respiratory functions is a challenge for physicians, and it is very important to reach a deeper understanding of this topic.

A caregiver’s distress level has been previously studied [24,25], but, as far as we know, none of these have investigated the relation between a caregiver’s psychological characteristics and clinical aspects of ALS patients. Our study is the first attempt to evaluate the interaction between psychological features of caregivers and their patient’s clinical data.

According to the obtained results, respiratory function is related to a patient’s perception of social support and to the psychological distress of their caregivers. In particular, the patient’s FVC and PCF present negative correlations with their caregiver’s depression, proneness to anxiety, and burden and a positive relation with perceived social support. Low levels of depression, trait anxiety, and burden experienced by caregivers correspond to higher values of FVC and PCF.

A first possible explanation for these results is that the clinical status of an ALS patient has a great influence their caregiver’s psychological well-being. It is quite intuitive, according to clinical experience, that the worsening of the physical condition of ALS an patient produces worries and burden to their relatives and close friends, in accordance with previous literature [9,26]. Considering the correlational design of the study, however, another possible explanation cannot be excluded, even if counterintuitive: Change can be promoted by an improvement in the quality of assistance, paying more attention to oral care, cough assistance, and secretion management. Relatively unworried caregivers may be able to provide a better assistance to their relatives by paying more attention to their needs, thus promoting better health condition for ALS patients. Conversely, high levels of depression, trait anxiety, and burden of caregivers could have had a negative effect on the quality of care in our sample. Previous findings by Chio and colleagues indicate that married ALS patients, after NIV, lived statistically longer than non-married [27]. These works, together with the present results, lead to the intriguing hypothesis that there could be something in the patient-caregiver relationship that influences the patient’s physical condition. Our data do not provide real empirical support to the hypothesis, but this issue should be addressed by future studies.

This small study has some limitations. No causal inferences between variables can be made in cross-sectional studies. Moreover, the relatively small sample size increases the risk of a type-II error in the interpretation of data. However, correlations are quite high, augmenting the validity of our results. Furthermore, the average FVC and PFC values were relatively high for ALS patients, although the results can not be generalized to all ALS subjects. The use of the Trait form of the STAI provides information about a subject’s proneness to anxiety and not the experienced level of anxiety. Therefore, more information about state anxiety will be required in future studies. Another limitation is that the sample includes only patients and caregivers with absent psychopathology prior to the ALS diagnosis. Dedicated works could address the clinical peculiarities of people with ALS and a previous psychopathology.

Even if the hypothesis of a partial causal relation between the caregiver’s psychological well-being and the patient’s respiratory function could be wrong, the importance of the caregiver’s role and social support should not be underestimated by physicians who work with ALS patients, considering the impact on the patient’s quality of life [28,29]. Together with patients, medical staff could take care of their closest relatives, providing psychological and social support. However, these kind of integrated treatment would seem not to be practical in most ALS clinics, which are already stretched to provide all the services the patients need.

In order to increase the scientific knowledge aimed at the improvement of clinical practice, further works are required to investigate the complex relationship between caregiver psychological characteristics and clinical course of ALS. Moreover, studies about possible psychological interventions are warranted in the ALS field [30]. We suggest the realization of a randomized clinical trail that compares patient-caregiver couples who receive psychological support with couples that do not, with both clinical and psychological outcomes.

## Conclusions

We found significant correlations between a caregiver’s distress, the perception of social support, and a patients’ respiratory function. The causal direction of these relations is unclear. Even if it seem probable that a caregiver’s burden is deeply influenced by an ALS patient’s physical condition, we also suggest another possible explanation, that perceived social support and the psychological well-being of a caregiver has a potential impact on the patient’s respiratory capacity. Along with this hypothesis, the management of anxiety, depression, and burden of ALS caregivers may have a positive effect on the patient’s respiratory condition through the improvement of home care.

More studies are required to investigate the interactions between the caregiver’s psychological characteristics and an ALS patient’s clinical features.

## Abbreviations

ALS: Amyotrophic Lateral Sclerosis; QoL: Quality of Life; FVC: Forced Vital Capacity; PCF: Peak Cough Flow; MQoL: McGill Quality of Life; MQOL-SS: Social Support” sub-scale of MQoL; ZBI: Zarit Burden Interview; BDI-II: Beck Depression Inventory-II; STAI: State-Trait Anxiety Inventory; SPSS: Statistical Package for Social Science.

## Competing interests

The authors declare that they have no competing interests.

## Authors’ contributions

FP designed and coordinated the study; PB, CL and MC provided the information about biomedical variables; GR, GC and EM supported the analysis and the interpretation of data; AM and FF supported the gathering of psychological information. All authors have read and approved the final manuscript.
